# Intravenous iron reactions: Insights from an allergy and immunology perspective

**DOI:** 10.1016/j.jacig.2025.100543

**Published:** 2025-07-21

**Authors:** Jordon Jaggers, Cosby Stone, Matthew Krantz, Elizabeth Phillips

**Affiliations:** a Department of Medicine, Center for Drug Safety and Immunology, Vanderbilt University Medical Center, Nashville, Tenn; b Institute for Immunology and Infectious Diseases, Murdoch University, Murdoch, Australia

**Keywords:** Adverse reactions, drug allergy, intravenous iron

## Abstract

**Background:**

Diagnosis and management of intravenous iron reactions is often challenging, as skin testing has unproven utility and most reactions are non–IgE-mediated.

**Objective:**

We aimed to identify clinical patterns and tolerability predictors in patients with reactions to intravenous iron.

**Methods:**

We conducted a retrospective cohort study of patients with reactions to intravenous iron who were referred to the Vanderbilt University Medical Center Drug Allergy Clinic from April 2014 through January 2025 and administered a follow-up survey via RedCap to evaluate patient outcomes with future intravenous iron administration.

**Results:**

Of the 51 patients presenting for adverse reactions to intravenous iron, 48 had skin testing performed. The skin testing results were deemed negative in all 48 cases (100%). Notable laboratory test results within 1 year of reaction were low vitamin D level (47%), high parathyroid hormone level (46%), and low phosphorus level (10%). Many patients (56%) were dermatographic, and their drug alert labels included opioids (31%), fluoroquinolones (14%), and radiocontrast dye (8%). Following assessment, 31 patients received intravenous iron (61%) using formulations that were the same as (n = 15 [48%]) and/or different from (n = 17 [55%]) the forumlation initially implicated, with the various modifications including antihistamines, slower infusion rate, and intravenous fluid pretreatment. Of these 31 patients, 27 (87%) tolerated the infusions. Additionally, following evaluation, the patients were surveyed regarding subsequent intravenous iron administrations, eliciting a 29% response rate (n = 15). Of the responders, 7 patients (47%) reported receiving intravenous iron after evaluation; all 7 reported tolerance.

**Conclusion:**

Most reactions to intravenous iron are non–IgE-mediated; however, our study introduces 2 novel observations, namely, a high frequency of dermatographism (56%) and common colabeling of these patients with drug alerts to Mas-related G protein–coupled receptor X2 (MRGPRX2)-activating drugs, suggesting a possible shared pathophysiologic mechanism.

## Introduction

The diagnosis and management of patients with reactions to intravenous iron (FEIV) who have been referred to allergists is challenging, as skin testing has unproven utility and most reactions are either non–IgE-mediated or nonallergic.^1^ Immediate (type 1) hypersensitivity reactions occur when IgE antibodies bind to the Fcε receptors on the surface of mast cells, causing cross-linking of cell-bound antibodies on mast cells and thus resulting in degranulation and release of vasoactive mediators such as histamines and leukotrienes.^2^ Most drugs and metals, including iron, are small molecules that alone would not result in a type 1 hypersensitivity reaction but would instead have to function as a hapten, a small molecule that could covalently bind to and alter a larger protein (a carrier), forming a hapten-carrier complex resulting a drug specific immune response.^2^^,^^3^ The Mas-related G protein–coupled receptor X2 (MRGPRX2) receptor is expressed on mast cells, and similar to type 1 hypersensitivity, it leads to degranulation and allergic-type inflammation.^4^ Notably, there are classes of medications that can activate the MRGPRX2 receptor on mast cells, resulting in degranulation and release of inflammatory mediators that in turn result in reactions appearing similar to IgE-mediated reactions, although they are not triggered by allergy-specific antibodies (IgE).^4^ These medications include fluoroquinolones, vancomycin, and some opioids, as well as some neuromuscular blocking agents and radiocontrast dye.^4^ Additionally, FEIV-induced hypophosphatemia, which is an often-underrecognized adverse event, varies by formulation, may be persistent, and can present with myalgias, muscle weakness, heart failure, respiratory failure, and rhabdomyolysis.^5^^,^^6^ Reported rates vary, including 0.0% to 92.1% for ferric carboxymaltose and 0.0% to 40.0% for iron sucrose; however, phosphorus levels are not part of routine laboratory panels, and hypophosphatemia can go undiagnosed.^7^^,^^8^ We hypothesized that most FEIV reactions are non–IgE-mediated, are tolerable with future modifications, and may involve identifiable clinical predictors such as dermatographism and prior drug alerts.

## Results and discussion

### Methods

We conducted a retrospective cohort study in our clinic population over the course of 10 years of FEIV reactions; the cohort members were referred to the Vanderbilt University Medical Center (VUMC) Drug Allergy Clinic from April 2014 through January 2025. The study included the exploratory goals of identifying clinical patterns and tolerability predictors as well as specifically collecting data on patient characteristics, iron formulation, reaction history, testing, recommendations, future tolerance, and relevant laboratory values. Allergy labels were reclassified as alert labels where appropriate, as many allergy labels may represent drug intolerances; the total alert labels included FEIV intolerance label if present.^9^ Potential IgE-mediated cases were initially suspected on the basis of clinical history, features such as rapid symptom onset, and multisystem involvement (skin, respiratory, gastrointestinal), but this was later deemed unlikely owing to negative skin test results and favorable outcomes with subsequent modified infusions. We administered a follow-up survey via RedCap to evaluate patient demographics, recommendation interpretation, and tolerance of FEIV (including modifications utilized following evaluation if applicable). Information was analyzed and summarized with descriptive statistics and univariate tests. This study was approved by the VUMC institutional review board under protocol no. 161455.

### Clinical characterization

Among the 51 patients presenting with 63 FEIV reactions, 50 (98%) were women, the median age was 37 years (interquartile range [IQR] = 27), 30 (59%) self-reported as being of the White race, and 14 (27%) self-reported as being of the Black race (Table I). The causes of iron deficiency included menorrhagia (41%), gastrointestinal bleeding (24%), and malabsorption (16%). Most patients (67%) with FEIV reactions had a history of drug intolerance syndrome with a median of 4 alert labels (IQR = 6) and 3 drug-specific alert labels (IQR = 5). Common drug intolerances reported included opioids (31%), fluoroquinolones (14%), and radiocontrast (8%). Environmental (57%) and food (14%) allergies were present in select patients, with the medication lists including antihistamines in 51% of cases. Dermatographism, which is now routinely evaluated in our patient population, was present in many patients when assessed (n = 20 [56%]).

**Table I:** Demographics, reaction characteristics, skin testing results and recommendations, and management outcomes

Indicator	Value, no. (%)
Demographic characteristic (N = 51)	
Female∗	50 (98)
Age (y), median (IQR)	37 (27)
Ethnicity†	
White	30 (59)
Black	14 (27)
Hispanic	3 (6)
Asian	1 (2)
Iron deficiency etiology‡	
Menorrhagia	21 (41)
Gastrointestinal source	12 (24)
Malabsorption	8 (16)
Other conditions	
Dermatographism assessed	36
Dermatographism present	20 (56)
Dermatographism absent	16 (44)
Chronic urticaria	4 (8)
Ehlers-Danlos syndrome	1 (2)
Environmental allergy	29 (57)
Food allergy	7 (14)
Antihistamine on medication list	26 (51)
Other drug alerts	34 (67)
Total alert labels, median, IQR	4, 6
Drug alert labels, median, IQR§	3, 5
Reaction characteristic (n = 63) and assessment (n = 51)	
Intravenous iron formulations implicated‖,¶	
Iron sucrose	35 (56)
Iron dextran	12 (19)
Ferric carboxymaltose	7 (11)
Ferric derisomaltose	2 (3)
Ferric gluconate	2 (3)
Ferumoxytol	2 (3)
Initial infusion of specific formulation#	39 (62)
Immediate reaction (<6 h)	55 (87)
Delayed reaction (≥6 h)∗∗	5 (8)
Organ systems involved in reaction	
Cutaneous	26 (41)
Central nervous system	19 (30)
Cardiovascular	17 (27)
Musculoskeletal	17 (27)
Gastrointestinal	16 (25)
Respiratory	15 (24)
Treatment received	39 (62)
Antihistamines	30 (48)
Steroids	18 (29)
Epinephrine	8 (13)
Intravenous fluids	6 (10)
Phosphorus repletion	2 (3)
Reaction type	
Potential IgE-mediated (by history)††	38 (75)
Non–IgE-mediated‡‡	6 (12)
Possible hypophosphatemia	6 (12)
Unknown reaction	1 (2)
Skin testing results (n = 48),§§ relevant laboratory test results, and recommendations (n = 51)	
Formulations utilized‖‖	
Iron sucrose 0.02, 0.2, and 2 mg/mL	48 (100)
Ferric gluconate 0.25 and 12.5 mg/mL	46 (96)
Iron dextran 0.05, 0.5, and 5 mg/mL	33 (69)
Result of skin testing	
Negative (across all concentrations)	48 (100)
Suggestive of irritant reactions	14 (29)
Notable laboratory result within 1 year of reaction	
Vitamin D level assessed	36
Normal vitamin D level	18 (50)
Low vitamin D level	17 (47)
High vitamin D level	1 (3)
PTH level assessed	13
Normal PTH level	7 (54)
High PTH level	6 (46)
Phosphorous level assessed	21
Normal phosphorous level	19 (90)
Low phosphorus level	2 (10)
Recommendations¶¶	
Slow infusion rate	38 (75)
Premedicate with antihistamines	33 (65)
Premedicate with intravenous fluids	23 (45)
Utilize different formulation	16 (31)
Assess phosphorus and vitamin D levels	13 (25)
Use test dose procedure	8 (16)
Avoid##	2 (4)
Management outcomes (n = 51)	
Received subsequent FEIV∗∗∗	31 (61)
Tolerated†††	27 (87)
Formulation‡‡‡	
Same formulation§§§	15 (48)
Different formulation‖‖‖	17 (55)

*PTH*, Parathyroid hormone level.

∗ Male (n = 1).

† Other (n = 1), unknown ethnicity (n = 2).

‡ Unknown etiology (n = 7), etiology related to pregnancy (n = 2), and etiology related to inadequate dietary intake (n = 1).

§ Drug alert labels included opioids (n = 16), fluoroquinolones (n = 7), radiocontrast dye (n = 4), vancomycin (n = 0).

‖ Unknown formulation (n = 3).

¶ Patient experienced reactions to FEIV: μ = 1.2 (SD = 0.5) formulations.

# Subsequent infusion was performed (n = 19); whether subsequent infusion was performed is unknown (n = 5).

∗∗ Unknown reaction time (n = 3).

†† Although disproved by negative testing.

‡‡ Includes known adverse effects (immediate myalgias), delayed nonspecific symptoms (headache, gastrointestinal symptoms), extremity swelling, numbness, energy loss, and erythema around intravenous site.

§§ Testing of 3 patients was not completed: 2 had telemedicine visits and the reaction of 1 was believed to not be immunologically mediated, so testing was not completed at an in-person visit.

‖‖ Testing to available formulations at time of visit, including via skin prick and intradermal testing.

¶¶ Recommendations also included no restrictions (n = 3) and use of the least-irritating formulation (n = 2).

## Given concern for vascular injury, 1 subject in initial consultation and 1 subject in repeat evaluation following multiple instances of FEIV tolerance after initial evaluation.

∗∗∗ See Table II for patient characteristics of those not tolerating subsequent FEIV.

††† One patient tolerated multiple subsequent exposures after initial evaluation but then developed concern for vasculitis with foot discoloration and therefore told to avoid subsequent exposure.

‡‡‡ Formulations included iron sucrose (n = 15), ferric gluconate (n = 7), iron dextran (n = 7), ferric carboxymaltose (n = 3), ferric derisomaltose (n = 1). One patient received formulations similar to and different from the formulation eliciting the initial reaction.

§§§ The same formulations included iron sucrose (n = 10), ferric carboxymaltose (n = 2), ferric gluconate (n = 1), ferric derisomaltose (n = 1), and iron dextran (n = 1).

‖‖‖ The different formulations included iron dextran (n = 6), ferric gluconate (n = 6), iron sucrose (n = 5), and ferric carboxymaltose (n = 1). One patient received 2 formulations that were different from the initial formulation.

Reactions occurred to a variety of FEIV preparations, most commonly iron sucrose (56%), iron dextran (19%), and ferric carboxymaltose (11%) on initial (62%) or subsequent (30%) infusions; the reactions included immediate (<6 hours [87%]) and delayed-onset (≥6 hours [8%]) reaction (Table I). The organ systems involved included the cutaneous (41%), central nervous system (30%), cardiovascular (27%), musculoskeletal (27%), gastrointestinal (25%), and respiratory (24%) systems. Management (62%) involved emergency department care (27%) and included administration of antihistamines (48%), steroids (29%), epinephrine (13%), intravenous fluids (10%), and phosphorus (3%).

### Skin testing and management strategies

Skin tests were performed on 48 patients (94%) with available iron formulations (Table I), all of which uniformly yielded negative or irritant results (n = 48 [100%]).

After history taking, skin testing, and interpretation had been performed, reactions were deemed to be potential IgE-mediated on the basis of history, although this was subsequently disproved by negative testing results (75%), being non–IgE-mediated (12%), and possible hypophosphatemia (12%) based on review of the postvisit recommendations. Nonallergic examples included known adverse effects (immediate myalgias), delayed nonspecific symptoms (headache, gastrointestinal discomfort), extremity swelling, numbness, fatigue, and erythema at the intravenous injection site. Notable laboratory results for those with values within 1 year of reaction were low vitamin D level (47%), high parathyroid hormone level (46%), and low phosphorus level (10%). Visit recommendations were guided primarily by clinical history, not by the skin testing results; they included slower infusion rate (75%), premedication with antihistamines (65%) and intravenous fluids (45%), utilization of a different formulation (31%) and test dose procedure (16%), and evaluation of phosphorus and vitamin D levels (25%). The recommendations by providers were not standardized, and there was no standard policy regarding premedication. Because of concern for FEIV-induced vascular injury, 2 patients were told to avoid FEIV (4%) (one after initial evaluation and the other after a subsequent treatment attempt).

### Future tolerability and survey outcomes

Following Drug Allergy Center assessment, 31 patients received FEIV (61%) using formulations that were the same (n = 15 [48%]) and/or different (n = 17 [55%]) from those initially implicated with antihistamine pretreatment (n = 5 [16%]), antihistamine and slower infusion rate (n = 6 [19%]), slower infusion rate (n = 5 [16%]), slower infusion rate and intravenous fluid pretreatment (n = 3 [10%]), intravenous fluid pretreatment alone (n = 1 [3%]), and all 3 modifications (n = 9 [29%]). Of these 31 patients, 27 (87%) tolerated the infusions. The formulations utilized included iron sucrose (n = 15), ferric gluconate (n = 7), iron dextran (n = 7), ferric carboxymaltose (n = 3), and ferric derisomaltose (n = 1) (Table I). The 4 individuals who did not tolerate further FEIV despite modified infusion varied; they included 1 individual with only antihistamine pretreatment, 1 individual with only a different formulation utilized, 1 individual with antihistamine and fluid pretreatment at a slower rate, and 1 individual with a different formulation and fluid pretreatment (Table II). The mean hemoglobin levels were higher for those who did not go on to receive future FEIV (12.8 [SD = 1.3] g/dL vs 11.8 [SD = 2.15] g/dL; *P* = .056). During their visit, patients were additionally evaluated for other drug alert labels (n = 11) to as many as 2 additional drugs (n = 3), including penicillin (n = 8), sulfamethoxazole-trimethoprim (n = 1), gadobutrol (n = 1), unspecified radiocontrast agent (n = 1), cephalexin (n = 1), and ciprofloxacin (n = 1).

**Table II:** Characteristics of those not tolerating subsequent FEIV (n = 4 [13%])

Patient No.	Index formulation(s)	Subsequent formulation	Modifications	Subsequent symptoms	Relevant laboratory results
1	Iron dextran, ferric gluconate	Iron dextran	Antihistamines	Worsening chest pain in the setting of active chest pain	None
2	Iron sucrose, ferric carboxymaltose	Ferric gluconate	Different formulation	Pruritus, hives, and extremity swelling during infusion	Low phosphorus level Normal vitamin D level Normal PTH level
3	Ferumoxytol, iron sucrose	Iron sucrose	Antihistamines Fluids Slower rate	Extremity burning and swelling after infusion	Normal phosphorus level Low vitamin D level Normal PTH level
4	Iron sucrose	Ferric gluconate	Different formulation Fluids	Arthralgias, swelling, and migraine after infusion	Normal phosphorus level Low vitamin D level Elevated PTH level

Following Drug Allery Center evaluation, patients received a survey regarding the outcomes of subsequent FEIV administration; the survey had a 29% response rate (n = 15). For qualitative questions, we used 5-point Likert scales. Of the responders, 7 (47%) reported receiving FEIV after evaluation, with all of them reporting tolerance. Of those individuals, 4 reported pretreatment with antihistamines and 4 reported slower infusion rates (3 of whom reported both). Subjects (n = 9 [60%]) reported being confident or very confident that they would be able to tolerate FEIV in the future with use of a modified treatment plan (n = 4 [with 27% feeling unconfident or very unconfident). All patients who reported feeling very confident had successfully tolerated FEIV after evaluation. Subjects reported being unlikely or very unlikely (n = 6 [40%]) to have a reaction if they received future FEIV administrations (with 4 [27%] being very likely or likely and 5 [33%] being neutral). Two patients who responded “very likely” reported that they had received a recommendation to avoid FEIV. Subjects who responded “very willing” or “willing” (n = 8 [53%]) to receive FEIV in the future if recommended to do so by their doctor (with 2 [13%] feeling neutral and 3 [20%] being unwilling or very unwilling).

FEIV reactions have different clinical manifestations, but our work continues to support the idea that they are largely non–IgE-mediated or nonallergic in nature, suggesting that reactions resembling immediate hypersensitivity are not a contraindication to rechallenge to the same or different formulations in the future, without the need for desensitization. We suggest that future guidelines may consider deemphasizing skin testing in FEIV evaluation workflows, except in a research context, as its utility remains unproved. Hypophosphatemia remained both underrecognized and undermonitored, which is consistent with broader real-world practice. Despite diagnostic challenges, hypophosphatemia remains a treatable FEIV-related adverse event. Even mild findings reinforce the need for proactive phosphate monitoring, especially in cases involving ferric carboxymaltose, which represents an opportunity for potential improvement of clinical quality.^10^

Iron sucrose was the most implicated formulation in our study (in 56% of cases), although notably, it is one of the most commonly administered FEIV formulations at VUMC (accounting for 40% of administered FEIV doses since recording those data in the electronic medical record began in November 2017), followed by ferumoxytol (22%), iron dextran (19%), and ferric carboxymaltose (17%). Although rates of anaphylaxis in response to currently available FEIV products are very low, it has been noted that there is a 3- to 8-fold greater risk for iron dextran and ferumoxytol than for iron sucrose, which may be considered when selecting a formulation.^11^ Our cohort likely diverges from this risk hierarchy, as it reflects VUMC use patterns. We emphasize that although rare, immediate reactions to a first dose of iron sucrose are possible, and use patterns heavily influence our observed reaction distributions.

In support of a non–IgE-mediated hypersensitivity mechanism, our study suggests that most patients tolerate future FEIV with antihistamine pretreatment and slower infusion rates as well as utilization of different formulations. Tolerability rates were high across all modification strategies, supporting the general effectiveness of slower infusion and premedication treatment, although we acknowledge that sample size limitations preclude formal comparative modeling. Those individuals who did not tolerate subsequent intravenous iron with modifications may be reacting through a different pathway, such as labile iron, which may cause nonspecific oxidative stress and complement activation.^1^ Future directions include conducting more powered analytic studies. Although a formal multivariable model was not feasible in this study, we observed a nonsignificant trend toward higher hemoglobin levels among those who did not receive subsequent iron. Additionally, the survey findings should be interpreted cautiously, given the low response rate and the fact that the responding cohort may have been biased toward patients with better outcomes. Limitations also include temporal bias, as a limited amount of time had elapsed since the evaluation to assess future tolerance.

Intriguingly, a high proportion of patients (56%) were dermatographic, a finding not previously emphasized in iron hypersensitivity cohorts with negative skin testing results and also labeled with alerts to other drugs such as fluoroquinolones, radiocontrast dye, and opioids, which are known to be MRGPRX2-activating drugs, thus suggesting a possible shared pathophysiologic mechanism.^12^ Non–IgE-mediated reactions are frequently evaluated by the allergist and remain to be fully understood; however, we believe that this study provides further insight into this population, which may be reacting to FEIV through a MRGPRX2 pathway. Additionally, mast cell activation may be occurring via complement pathways rather than via classical IgE-mediated anaphylaxis, such as complement activation–related pseudoallergy triggered by iron nanoparticles as a possible mechanism in acute reactions.^13^ Our observations may further inform investigations searching for unifying pathways behind non–IgE-mediated mast cell activation, and they highlight the need for further studies to define specific mechanisms.

**Key messages**

• Intravenous iron reactions have different clinical manifestations but are largely non–IgE-mediated and nonallergic, suggesting that they are not absolute contraindications for future administration.
• Most subjects tolerate future administration of intravenous iron with antihistamine pretreatment and reduced infusion rates, thus enhancing safety and feasibility.
• A high frequency of dermatographism and common colabeling of drug alerts to MRGPRX2-activating drugs suggests a possible shared pathophysiologic mechanism for intravenous iron reactions.

## Disclosure statement

Supported by the 10.13039/100000133Agency for Healthcare Research and Quality (grant R01HS03023401 [to C.S.]), the 10.13039/100000060National Institute of Allergy and Infectious Diseases (grant U01AI181927 [to C.S.]), a 10.13039/100007208Vanderbilt-Ingram Cancer Center/Chic Awareness P3 Pilot Award that is unrelated to this article (to C.S.), the 10.13039/100019212AAAAI Foundation (to M.S.K.), the 10.13039/100000002National Institutes of Health (grant K08AI185260 [to M.S.K.] and grants R01HG010863, R01AI152183, and U01AI154659 [all to M.S.K.]), and the Australian 10.13039/501100000925National Health and Medical Research Council. In addition, E.P. receives grant funding as a principal investigator from the 10.13039/100000002US National Institutes of Health.

Disclosure of potential conflict of interest: E. Phillips has received funding from Janssen, Verve, Rapt, Servier, and Esperion, as well as royalties and consulting fees from UpToDate. The rest of the authors declare that they have no relevant conflicts of interest.
